# Diagnostic accuracy of Panbio™ rapid antigen test for SARS-CoV-2 in paediatric population

**DOI:** 10.1186/s12887-023-04201-z

**Published:** 2023-08-29

**Authors:** Laura Gallardo-Alfaro, Patricia Lorente-Montalvo, Margarita Cañellas, Eugenia Carandell, Antonio Oliver, Estrella Rojo, Beatriz Riera, Joan Llobera, Oana Bulilete, on behalf of Balearic COVID-19 Pediatric Primary Care Research Group, Alfonso Leiva, Anaida Obieta, Victoria Pascual, Pau Pericas, Carlos Radúan, Elsa Segura, Verónica Vega

**Affiliations:** 1 Primary Health Care Research Unit, Balearic Public Health Service (Ib-Salut), Escola Graduada n9, 07002 Palma, Illes Balears Spain; 2 Balearic Islands Health Research Institute (IdISBa), Carretera de Valldemossa, 79, 07120 Palma, Illes Balears Spain; 3 RICAPPS- Red de Investigación Cooperativa de Atención Primaria Y Promoción de La Salud - Carlos III Health Institute (ISCIII), Madrid, Spain; 4 Santa Ponça Primary Health Care Center, Balearic Public Health Service (Ib-Salut), Carrer del Riu Síl, 25, 07180 Santa Ponça, Illes Balears Spain; 5 Son Llatzer University Hospital, Ctra. de Manacor, 07198 Palma, Illes Balears Spain; 6 Directorate of General Health Service, Balearic Public Health Service (Ib-Salut), Reina Esclaramunda n9, 07005 Palma, Illes Balears Spain; 7 Microbiology Service, Son Espases University Hospital, Balearic Public Health Service (Ib-Salut), Carretera de Valldemossa, 79, 07120 Palma, Illes Balears Spain

**Keywords:** COVID-19, Rapid antigen test, SARS-CoV-2, Primary care, Paediatric

## Abstract

**Background:**

Rapid antigen-detection tests (Ag-RDTs) are used to diagnose SARS-CoV-2 infection. Real-world studies of Ag-RDTs are necessary to evaluate their diagnostic yield in paediatric patients. Our aim was to evaluate the accuracy of the Panbio™ Rapid Antigen Test for SARS-CoV-2 in the setting of a primary health care centre (PHC), with use of the Reverse Transcription-Polymerase Chain Reaction (RT-PCR) as gold standard.

**Methods:**

This prospective diagnostic study was conducted at PHCs in Mallorca, Spain. Patients were ≤ 18 years-old that attended sites for RT-PCR testing due to symptoms suggestive of infection (fever, headache, nasal congestion and dry cough, among others) or epidemiological exposure (close contacts). Two samples were collected: a nasal mid-turbinate sample for Ag-RDTs and a nasopharyngeal swab for RT-PCR testing. The sensitivity, specificity, and predictive values of the AgRDT were calculated using the RT-PCR results as the reference.

**Results:**

We examined 1142 participants from 0 to 18 years (47.5% female, mean age 8.9 ± 4.8 years, median 9.0 [5.0–13.0]). There were 84 positive RT-PCR results (pre-test probability of 7.3%) and 52 positive Ag-RDT results. The sensitivity of the Ag-RDT was 59.5% (95% Confidence Interval (CI): 48.2–69.9%), the specificity was 99.8% (95%CI: 99.2–99.9%), the positive predictive value was 96.1% (95%CI: 85.6–99.4%), and the negative predictive value was 96.8% (95%CI: 95.6–97.7%). The sensitivity for individuals referred by a general practitioner (GP) or paediatrician due to symptoms was 71.4% (95%CI: 51.5–86.0%) and for asymptomatic individuals was 50.0% (95%CI: 9.1–90.8%). The specificity was greater than 98.9% overall and in all subgroups. The sensitivity was 73.0% (95%CI: 52.0–87.5%) for referred patients due to symptoms and who were tested within 5 days since symptom onset. No significant statistical differences between any groups were found. There were 34 false-negative Ag-RDT results (40.5%) and 2 false-positive Ag-RDT results (0.2%).

**Conclusion:**

The sensitivity of the Panbio™ Test in paediatric individuals is below the minimum of 80% recommended by the World Health Organization for Ag-RDTs. This test had better accuracy in individuals referred by a GP or paediatrician due to symptoms, rather than those who were asymptomatic or referred due to epidemiological exposure. The RT-PCR test using a nasopharyngeal swab is accurate, but a less invasive alternative that has better sensitivity than the Panbio™ Test is needed for paediatric populations.

## Introduction

Healthcare systems worldwide were severely strained by the rapid spread of severe acute respiratory syndrome coronavirus 2 (SARS-CoV-2). In general, detection and management of a growing surge of infections requires diagnostic tests that are easy to use and produce rapid results. Subpopulations, such as patients with asymptomatic infections and children, are poorly represented in studies that examine the accuracy of diagnostic tests [1].

Since the beginning of the COVID-19 pandemic, laboratories have used nucleic acid amplification tests, such as real-time reverse transcription polymerase chain reaction (RT-PCR) assays, to detect SARS-CoV-2, but these tests are more laborious and time-consuming than the rapid antigen-detection tests (Ag-RDTs) [2]. Ag-RDTs are designed to directly detect SARS-CoV-2 antigens produced by the replicating virus in the respiratory tract using point-of-care testing. After collecting a respiratory specimen and performing the test, the results are typically available within 30 min. The World Health Organization (WHO) recommended the use of Ag-RDTs that meet the minimum requirements of 80% sensitivity and 97% specificity [2, 3].

Previous studies showed that the sensitivity of RT-PCR tests from the upper respiratory tract (nasal or nasopharyngeal swabs) was highly variable. In addition, a 2021 Cochrane systematic review of 48 studies using Ag-RDTs reported a notable difference in the sensitivity for symptomatic adults (72.0%, 95% CI: 63.7–79.0%) and asymptomatic adults (58.1%, 95% CI: 40.2–74.1%) [1]. A July 2022 Cochrane systematic review analysed 152 studies and reported higher variations in sensitivity for symptomatic adults (73.0%, 95% CI: 69.3.2–76.4%) than asymptomatic adults (54.7, 95% CI: 47.7–61.6%), but consistently high specificity [4].

More recent studies have reported improved accuracy of Ag-RDTs, specially for patients with high viral loads [5]. A 2021 study of the Panbio™ Rapid Antigen Test with nasopharyngeal swabs in Mallorca (Spain) examined 1369 participants (≥ 18-years-old), reporting a sensitivity of 71.4%, being higher in symptomatic patients who were tested within 5 days since the onset symptoms (80.4%) [6]. Dinnes et al*.* (2022) compared the sensitivity in children and adults and found that the average sensitivity was 9.9% higher in adults, although this difference was not statistically significant [4]. Although some studies using Ag-RDTs obtained high sensitivity and specificity, there are typically small numbers of cases in certain subpopulations, making it difficult to form definitive conclusions regarding test accuracy in these groups [7]. For example, one study conducted in Spain examined 412 patients who visited Primary Care Health Centres (PHCs) and emergency wards, but only included 85 children (< 16 years-old). They found the sensitivity was higher in adults (82.6%, 95% CI: 63.3–90.9%) than children (62.5%, 95% CI: 30.6–86.3%) [8]. Gonzalez-Donapetry et al*.* (2021) examined 440 nasopharyngeal swabs taken from children at a hospital paediatric emergency department, and found the overall sensitivity was 77.7% and specificity was 100% [9], whereas Villaverde et al*.* [10] examined 1620 symptomatic children and reported a sensitivity of 45.4% (95% CI: 34.1–57.2%).

Sample collection is a critical factor affecting the performance of any diagnostic test that examines respiratory fluids, especially in children. Alemany et al*.* (2021) [11] obtained adequate results from less invasive samples (nasal samples, saliva samples, or both) in symptomatic and asymptomatic subjects. Moreover, Spanish authorities provided a provisional authorization for the Panbio™ Test in November 2020, and the manufacturer reported a sensitivity of 98.1% and a specificity of 99.8% [12]. However, there is a need for additional studies conducted in PHCs with large samples of children to establish the accuracy of Ag-RDT in paediatric populations. In particular, children are more likely to accept mid-turbinate sampling than nasopharyngeal sampling.

The main aim of this study was to evaluate the accuracy of the Panbio™ Test when used at a PHC in children who are symptomatic or had close contact with an infected person, with the RT-PCR test used as the gold standard.

## Methods

### Design and setting

This prospective diagnostic study was conducted from February to April 2021 in Mallorca (Balearic Islands, Spain) at different locations in the cities of Palma and Inca. There were 1023 participants recruited at a PHC testing location and 119 at the Emergency Department of the Son Espases University Hospital.

### Study population

All participants were 18 years-old or younger, unvaccinated, and attended one of the above-named settings for RT-PCR testing. All of them had symptoms suggesting infection, who were referred by a general practitioner (GP), or were exposed to another patient who had an infection confirmed by RT-PCR (close contact), who were referred by a specific COVID-19 call centre.

### SARS-CoV-2 testing

Trained health professionals collected all samples. First, a nasal mid-turbinate sample (for the Ag-RDT) was collected by inserting the swab about 2 cm into each nostril and rotated after insertion. Then, a nasopharyngeal sample (for the RT-PCR test) was collected by deep insertion of a swab into a single nostril and rotated.

### RT-PCR

Within 24 h of sample collection, the nasopharyngeal swab was sent for processing at the Microbiology Department of Son Espases University Hospital. The lab technicians performing the RT-PCR tests were blinded to the results of the Ag-RDTs and all additional information about the participants. RNA extraction was performed using the MagMAX™ Viral/Pathogen Nucleic Acid Isolation Kit (ThermoFisher) and amplification was performed using the TaqPath™ COVID-19 CE-IVD RT-PCR Kit and QuantStudio™ (ThermoFisher). The relative viral load was expressed as the cycle threshold (Ct) for the open reading frame (ORF), nucleocapsid (N), and spike (S) genes. High viral load was considered as a Ct below 25, a moderate viral load as a Ct of 25 to 29.9, and a low viral load as a Ct above 30 [2].

### Rapid antigen test

The nasal mid-turbinate swab was analysed on-site using the Panbio™ Test COVID-19 Ag Rapid Test Device (nasal) (Abbott Diagnostics GmbH, Germany), and the results were interpreted within 15 min as described by the manufacturer. The sample on the swab was mixed in 300 µL of buffer, and then 5 drops were dispensed onto the device. This chromatographic test contains an immobilized anti-SARS-CoV-2 antibody that binds to the N protein on the test line and a mouse monoclonal anti-chicken IgY on the control line. The two conjugates (a human IgG specific to a SARS-CoV-2 Ag gold conjugate that binds the N protein [test] and a chicken IgY gold conjugate [control]) move upward on the membrane and react with their corresponding antibodies. Neither the control line nor the positive test line was visible in the result window before adding the specimen. After 15 min, a visible control line indicated the test was valid and a visible second line indicated a positive result.

### Questionnaire

Participants answered a short questionnaire that asked about the reasons for the RT-PCR testing, socio-demographic information (gender and age), presence and type of symptoms, and number of days since symptom onset or epidemiological exposure to a positive patient.

### Statistical analysis

To evaluate the accuracy of the Panbio™ Test, the initial pre-test probability was 12.7% for symptomatic patients and 7.3% for asymptomatic patients. The sample size was calculated according to a sensitivity of 85% and a precision of 10% for symptomatic patients, and a sensitivity of 70% and a precision of 15% for asymptomatic patients. Thus, it was necessary to test 880 participants.

The sensitivity, specificity, and their 95% CIs of the Ag-RDT were calculated using the RT-PCR results as the reference. Sensitivity analysis was also performed by stratification according to the declared reason for RT-PCR testing, the presence of symptoms, days since symptom onset or epidemiological exposure, and Ct-values. Predictive values and 95% CIs were estimated using the pre-test probability results for each analysed group. Means and standard deviations were used for descriptive analysis of the study populations. Interaction testing through a binary logistic regression model was used to determine the statistical significance differences between groups (symptomatic vs. asymptomatic; tested within 5 days or > 5 days; according to the viral load). All statistical calculations were performed using SPSS statistical software version 25.0 (SPSS Inc., Chicago, IL, USA).

## Results

### Characteristics of patients

**Fig. 1:**
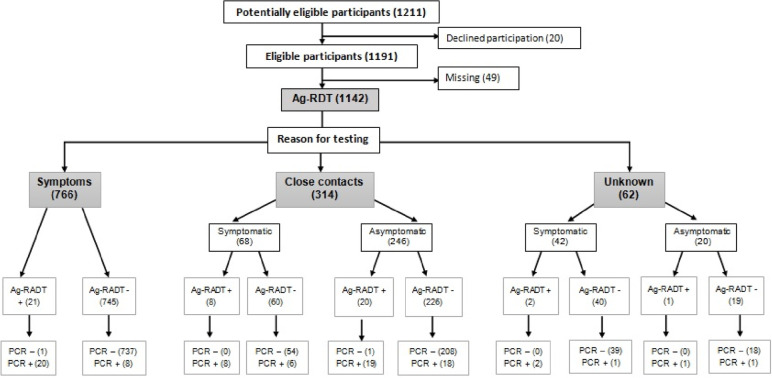
Enrolment and clinical characteristics of children who received RT-PCR testing and the Panbio™ Test

We initially identified 1211 potentially eligible participants (Fig. 1). Twenty individuals (1.7%) declined participation, mostly because the perceived discomfort caused by collection of two samples. Among the 1191 eligible participants, 49 (4.1%) were lost to follow up due to missing results. The final study population consisted of 1142 participants, the mean age was 8.9 ± 4.8 years, and 47.5% were female. The overall pre-test probability of COVID-19 was 7.5%, and there were 84 positive RT-PCR tests and 52 positive Ag-RDT results.

At enrolment, we recorded the demographic and clinical characteristics of all participants (Table 1). The main reasons for testing were referral by a GP or paediatrician due to symptoms compatible with COVID-19 and epidemiological exposure to an individual who had RT-PCR-confirmed positivity. A small number of other individuals were referred by a GP with no declaration of the reason (*n* = 62). The majority of individuals reported symptoms (76.3%), with nasal congestion (30.2%), fever (27.6%), dry cough (27.1%), headache (26.4%), sore throat (21.0%), and abdominal pain (17.9%) being the most prevalent. A high proportion of participants (86.9%) were tested within 5 days of the onset of symptoms or epidemiological exposure.

**Table 1:** Demographic and clinical characteristics of enrolled patients

	**N (%)**	**RT-PCR + (N,%)**	**Ag-RDT + (N,%)**
**Entire sample**	1142 (100)	84 (7.4)	52 (4.6)
**Age (mean ± SD)**	8.9 ± 4.8	10.8 ± 4.9	10.4 ± 5.0
< 5 years	277 (24.3)	11(13.1)	7 (13.59
5–9 years	309 (27.1)	20 (23.8)	15(28.8)
10–14 years	408 (35.7)	29 (34.5)	15(28.8)
15–18 years	148 (13.0)	24 (28.6)	15(28.8)
**Sex**			
Female	543 (47.5)	37(44.0)	21(40.4)
Male	591 (51.8)	47(56.0)	31(59.6)
Unknown	8 (0.7)		
**Reason for testing**			
Symptoms	766 (67.1)	28(33.3)	21(40.4)
Close contact	314 (27.5)	51(60.7)	28(53.8)
Unknown	62 (5.4)	5(6.0)	3(5.7)
**Declared symptoms**			
Yes	876 (76.7)	45(53.6)	31(59.6)
No	266 (23.3)	39(46.4)	21(40.4)
Fever	315 (27.6)	12(14.3)	9(17.3)
Dry cough	309 (27.1)	13(15.5)	8(15.4)
Sore throat	240(21.0)	8(9.5)	6(11.5)
Chest pain	22(1.9)	2(2.4)	1(1.9)
Shortness of breath	61 (5.3)	2(2.4)	1(1.9)
Abdominal pain	204(17.9)	8(9.5)	6(11.5)
Conjunctivitis	4 (0.4)	1(1.2)	1(1.9)
Muscle/joint pain	32(2.8)	3(3.6)	1(1.9)
Headache	301(26.4)	24(28.6)	18(34.6)
Diarrhoea	153(13.4)	8(9.5)	5(9.6)
Nasal congestion	345 (30.2)	15(17.9)	13(25.0)
Loss of smell	8 (0.7)	4(4.8)	3(5.8)
Loss of taste	11 (1.0)	3(3.6)	2(3.8)
Skin involvement	20 (1.8)	0 (0)	0(0)
Aches and pains	63 (5.5)	6(7.1)	6(11.5)
Neurologic symptoms	58 (5.1)	3(3.6)	3(5.8)
Other^a^	114(10.0)	7(8.3)	6(11.5)
Not known	1(0.1)	0(0)	0(0)
**Days since SO/CC**			
≤ 5	992 (86.9)	75(89.3)	46(93.9)
> 5	95 (8.3)	4(4.8)	3(6.1)
Unknown	55 (4.8)	5(6.0)	
**Viral load (Ct)**			
N gene (mean ± SD)	21.9 ± 6.5	23.4 ± 6.6	20.2 ± 6.1
S gene (mean ± SD)	24.3 ± 6.3	24.3 ± 6.8	21.3 ± 5.5
ORF gene (mean ± SD)	21.5 ± 6.7	23.1 ± 6.7	19.8 ± 6.4
N gene: Ct < 25	49(60.5)	49(58.3)	36(69.2)
N gene: Ct = 25.0–29.9	22(27.29	22(26.2)	9(17.3)
N gene: C ≥ 30.0	10(12.3)	10(11.9)	3(5.8)

*Abbreviations*: *RT-PCR* Reverse transcription polymerase chain reaction, *Ag-RDT* Rapid antigen diagnostic test, *SO* Symptom onset, *CC* Close contact, *Ct* RT-PCR cycle threshold

^a^Including tiredness, decreased food intake, lack of appetite, low-grade fever, dizziness, and eye discomfort

Among all patients with usable Ct values, the mean Ct was 21.9 ± 6.5 for the N gene, 24.3 ± 6.3 for the S gene, and 21.5 ± 6.7 for the ORF gene (Table 1). More than 60% of the participants with available Ct values had high viral loads for the N gene. The dominant SARS-CoV-2 variant was B.1.177, although 38 patients (45.2% of the positive samples) had infections by the B.1.1.7 variant.

### Overall test accuracy

We then determined the accuracy of the Panbio™ Test (Table 2). Based on the RT-PCR results, the pre-test probability was 7.3%. For all 1142 patients, the Panbio™ Test had a sensitivity of 59.5% (95% CI: 48.2–69.9%), a specificity of 99.8% (95% CI: 99.2–99.9%), a positive predictive value (PPV) of 96.1% (95% CI: 85.6–99.4%), and a negative predictive value (NPV) of 96.8% (95% CI: 95.6–97.7%).

**Table 2:** Overall accuracy of the Panbio™ Test

	**Prevalence** (%)	**Sensitivity** % (95% CI)	**Specificity** % (95% CI)	**PPV** % (95% CI)	**NPV** % (95% CI)
**Overall** (*N* = 1142)	7.3	**59.5** (48.2,69.9)	**99.8** (99.2,99.9)	**96.1** (85.6,99.4)	**96.8** (95.6,97.7)
**Overall: reason for testing**					
GP referral for symptoms (*N* = 766)	3.6	**71.4** (51.1,86.0)	**99.8** (99.1,99.9)	**95.2** (74.1,99.7)	**98.9** (97.8,99.4)
Close contact (*N* = 314)	16.2	**52.9** (38.5,66.8)	**99.6** (97.5,99.9)	**96.4** (79.7,99.8)	**91.6** (87.6,94.4)
Unknown (*N* = 62)	8.7	**60.0** (17.0,92.7)	**100.0** (92.1,100.0)	**100.0** (31.0,100.0)	**96.6** (87.3,99.4)
**Overall: symptoms**					
Yes (*N* = 876)	5.1	**66.7** (51.8,81.6)	**99.9** (99.6,100.0)	**96.8** (88.9,100.0)	**98.2** (97.3,99.2)
No (*N* = 266)	14.7	**51.3** (34.3,68.3)	**99.6** (98.5,100.0)	**95.2** (83.8,100.0)	**92.2** (88.7,95.8)

*Abbreviations*: *CI* Confidence interval, *GP* General practitioner, *PPV* Positive predictive value, *NPV* Negative predictive value

**Fig. 2:**
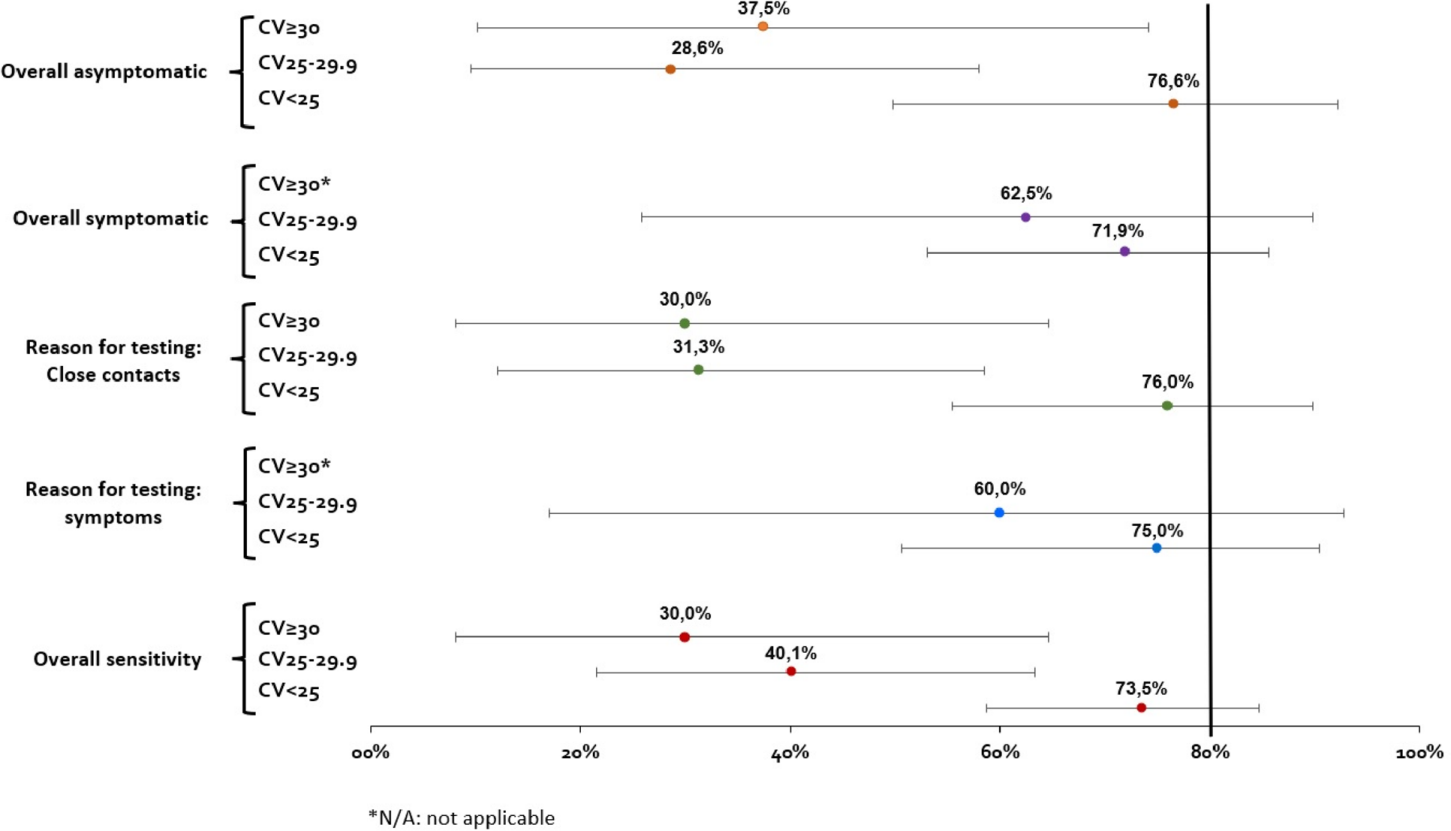
Sensitivity of the Panbio™ Test in our paediatric population with different clinical status and N gene viral load

Regarding the reason for testing, individuals referred by a physician due to symptoms (*n* = 766) compared to close contacts (*n* = 314), showed better sensitivity (71.4%, 95% CI: 51.5–86.0% *vs.* 52.9%, 95% CI: 38.5–66.8%) and specificity (99.8%, 95% CI: 99.1–99.9% *vs.* 99.6%, 95% CI: 97.5–99.9%), *p-value* = 0.69. Furthermore, the test sensitivity was higher for individuals who had symptoms (*n* = 876; 66.7%, 95% CI: 51.8–81.6%) than for those who were asymptomatic (*n* = 266; 51.3%, 95% CI: 34.3–68.3%), *p-value* = 0.19. The test specificity was greater than 98.9% overall and in all subgroups. The sensitivity according to the viral N gene load (Fig. 2) was higher (73.5%, 95% CI: 49.8–92.4%) in patients with high viral loads (Ct < 25), *p-value* = 1.00. Sensitivity in all the comparisons was not statistically significant.

### Test accuracy for patients tested at different times and had different reasons for testing

Table 3 shows the accuracy of the Panbio™ tested at different times according to reasons for testing or the presence/absence of symptoms. Most participants (86.9%) were tested within 5 days since symptom onset or epidemiological exposure, and the overall test sensitivity in this subgroup was 60.0% (95% CI: 48.0–70.9%). For all asymptomatic participants tested within 5 days of exposure or referral, the sensitivity was 48.5% (*n* = 205; 95% CI: 31.7–65.7%). However, the test sensitivity was higher for those who reported symptoms and was highest for those referred by a GP or paediatrician due to symptoms. The test sensitivity was low for close contacts. Analysis of the subgroup that was tested more than 5 days since symptom onset or epidemiological exposure indicated the overall test sensitivity in patients with or without symptoms was 50.0% (*n* = 95; 95% CI: 9.1–90.8%). No statistically significant differences were found between those tested within 5 days compared to those tested > 5 days, *p-value* = 0.12.

**Table 3:** Accuracy of the Panbio™ Test in patients tested 5 or fewer days (top) or more than 5 days (bottom) since symptom onset or close contact

	**Prevalence** (%)	**Sensitivity** (%, 95% CI)	**Specificity** (%, 95% CI)	**PPV** (%, 95% CI)	**NPV** (%, 95% CI)
** ≤ 5 days**					
Overall (*N* = 992)	**7.5**	**60.0** (48.0,70.9)	**99.8** (99.2,99.9)	**97.8** (87.0,99.8)	**96.8** (95.4,97.8)
**Overall symptoms**					
Symptomatic (*N* = 787)	**5.1**	**70.0** (53.2,82.9)	**99.8** (99.1,99.9)	**96.5** (80.3,99.8)	**98.4** (97.1,99.1)
Asymptomatic (*N* = 205)	**16.6**	**48.5** (31.7,65.7)	**100.0** (97.3,100.0)	**100.0** (77.0,100.0)	**90.6** (85.4,94.2)
**Overall: reason for testing**					
GP referral for symptoms (*N* = 692)	**3.8**	**73.0** (52.0,87.5)	**99.8** (99.0,100.0)	**95.0** (73.1,99.7)	**99.0** (98.0,99.5)
Overall close contacts (*N* = 250)	**18.0**	**51.1** (36.0,66.1)	**100.0** (97.7,100.0)	**100.0** (82.2,100.0)	**90.3** (85.5,93.7)
Symptomatic close contacts (*N* = 59)	**20.3**	**58.3** (28.6,83.5)	**11.9** (5.3,23.5)	**100.0** (56.1,100.0)	**90.4** (78.2,96.4)
Asymptomatic close contacts (*N* = 191)	**17.3**	**48.5** (31.2,66.2)	**100.0** (97.0,100.0)	**100.0** (75.9,100.0)	**90.3** (84.7,94.1)
** > 5 days**					
Overall (*N* = 95)	**4.2**	**50.0** (9.1,90.8)	**98.9** (93.1,99.9)	**66.6** (12.5,98.2)	**97.8** (91.6,99.6)
**Overall symptoms**					
Overall symptomatic (*N* = 69)	**2.9**	**50.0** (2.7,97.3)	**100.0** (93.2,100.0)	**100.0** (5.4,100.0)	**98.5** (91.0,100.0)
Overall asymptomatic (*N* = 26)	**7.7**	**50.0** (2.7,97.3)	**95.8** (76.9,99.8)	**50.0** (2.7,97.3)	**95.8** (76.9,99.8)
**Overall: reason for testing**					
GP referral for symptoms (*N* = 60)	**1.7**	**0.0** (0.0,94.5)	**100.0** (92.4,100.0)	N/A	N/A
Overall close contacts (*N* = 33)	**9.1**	**66.7** (12.5,98.2)	**96.7** (81.0,99.8)	**66.7** (12.5,98.2)	**96.7** (81.0,99.8)
Symptomatic close contacts (*N* = 8)	**12.5**	**100.0** (5.4,100.0)	**100.0** (56.1,100.0)	**100.0** (5.4,100.0)	**100.0** (56.1,100.0)
Asymptomatic close contacts (*N* = 25)	**8.0**	**50.0** (2.7,97.3)	**95.7** (76.0,99.8)	**50.0** (2.797.3)	**95.7** (76.0,99.8)

*Abbreviations*: *CI* 95% confidence interval, *GP* General practitioner, *N/A* Not applicable, *PPV* Positive predictive value, *NPV* Negative predictive value

### False-negative and false-positive results

Overall, there were 34 false-negative Ag-RDT results (40.5%) among the 84 patients who were positive in the PCR-RT test. These individuals were mainly 10 to 14 years-old and about half of them were females (47.1%). Analysis of these 34 patients indicated that 24 (70.6%) had epidemiological exposure to an infected individual and 19 (55.9%) were asymptomatic. There were false-positive Ag-RDT results in only 2 of 1056 patients (0.2%), an asymptomatic 5 year-old male who was a close contact and was referred more than 5 days since epidemiological exposure and a symptomatic 7 year-old male with dry cough, nasal congestion, sore throat and headache, who was referred by the GP within 5 days since symptoms onset.

## Discussion

We assessed the accuracy of the Panbio™ Test for SARS-CoV-2 in a paediatric population. During the 3 months study period, most patients were referred to PHCs because they had close contact with individuals who had confirmed COVID-19 infections or had symptoms consistent with COVID-19. The major result of this study was that this test had the highest sensitivity when an individual was referred by a GP or paediatrician and when testing was performed within 5 days since the onset of symptoms or epidemiological exposure, although no statistically significant differences were found. It is also important that the specificity of this test was above 98.9% overall and in all subgroups. The WHO recommended that Ag-RDTs for SARS-CoV-2 have sensitivity of at least 80% and specificity of at least 97% [2, 3]. Thus, in our paediatric population the Panbio™ test had acceptable specificity but unacceptable sensitivity.

A 2021 meta-analysis that assessed the diagnostic accuracy of Ag-RDTs for COVID-19 in paediatric populations under real-world conditions concluded that the performance of the current Ag-RDTs varied broadly among children [13]. Other studies showed that the sensitivity of the Ag-RDTs was higher in adults than children [4, 7, 9]. Gonzalez-Donapetry et al*.* [9] also tested nasopharyngeal swabs in a paediatric population using the Panbio™ Test and reported a sensitivity of 77.7%, with a lower value in children than adults. Eliseo et al*.* [8] reported a sensitivity of 62.5% (95% CI: 30.6–86.3%) in children (*n* = 412) in PHC. Villaverde et al*.* [10] examined 1620 symptomatic children and reported a sensitivity of 45.4% (95% CI: 34.1–57.2%). Carbonell-Sahuquillo et al*.* [14] examined 357 patients at a paediatric emergency department and reported this test had a sensitivity of 70.5%. Möckel et al*.* reported a sensitivity of 72.0% (95% CI: 53.3–86.7%) in paediatric cohort (*n* = 202) in an emergency department [15]. Pollock et al*.* used a similar Ag-RDT (BinaxNOW) that uses anterior nasal swabs and reported a lower sensitivity in children than in adults at the hospital; the sensitivity was 84.6% (95% CI: 65.1–95.6%) in symptomatic children and 65.4% (95% CI: 55.6–74.4%) in asymptomatic children [16]. Sood et al*.* also used the BinaxNOW test (*n* = 226) and reported a sensitivity of 56.2% (95% CI: 49.5–62.8%) [17]. Thus, Ag-RDTs appear to be unsuitable for testing an entire asymptomatic population, a practice performed early during the pandemic in some countries [18], in which testing was performed in locations such as airports, train stations, and schools [19]. In contrast, the sensitivity was 70.0% (95% CI: 53.2–82.9%) in our symptomatic children, and was slightly higher when the child was referred by a GP for symptoms compatible with COVID-19 (73.0%, 95% CI: 52.0–87.5%). This result highlights the importance of these professionals in a real-world setting. Furthermore, a possible explanation for the lower sensitivity in children than adults in the present study and in previous studies may be the difficulty in collecting nasal samples from children. Sample collection is one of the most important factors affecting the performance of any diagnostic test [3].

Notably, other SARS-CoV-2 tests also showed lower sensitivity for asymptomatic than symptomatic individuals [20]. Drêvínek et al*.* concluded that because Ag-RDTs have low sensitivity in asymptomatic individuals, they should be administered repeatedly at high frequency, and RT-PCR testing should be used for the most vulnerable populations [21]. This aligns with the study by Torres et al*.* [5], who concluded that the Panbio™ Test had low sensitivity in asymptomatic close contacts of COVID-19 patients. In 2021, the WHO concluded that Ag-RDTs should be prioritized for symptomatic individuals who meet the case definition for COVID-19 and asymptomatic individuals who have high risk of infection. Moreover, it must be considered that the Ag-RDTs are less sensitive than the RT-PCR test, especially in asymptomatic patients [3]. In contrast, Polechová et al. recommended use of Ag-RDTs at least 2 to 3 times per week as a large-scale Ag-RDT strategy for Austrian schools [22].

Only 95 of our children were tested more than 5 days after the onset of symptoms or epidemiological exposure, and the overall sensitivity in these children was only 50.0% (95% CI: 9.1–90.8%). This is in accordance with the results of He et al*.*, who concluded that transmission could potentially occur 2 to 3 days before symptom onset, but only 1% of all transmissions occurred within 5 days before symptom onset [23]. These results suggest that testing of paediatric populations should be prioritized for children within 5 days since symptom onset.

Notably, the overall pre-test probability in our study was only 7.3%. There is evidence that a lower disease prevalence leads to a lower PPV for Ag-RDTs [24, 25]. Nonetheless, we had a high overall PPV (96.1%, 95% CI: 85.6–99.4%) and a high overall NPV (96.8%, 95% CI: 95.6–97.7%).

Some studies showed that despite the high specificity of the Ag-RDTs, false positive results can occur, especially when testing a population with a low prevalence [3]. Although our pre-test probability was only 7.3%, so was our percentage of false positive results (0.2%). Our two false positive results were in children who were 5 years-old and 7 years-old. Similarly, L’Huillier et al. [26] reported a trend for lower Ag-RDT sensitivity in symptomatic children who were under 12 years-old relative to those who were 12 years-old or more.

Use of the RT-PCR test with a nasopharyngeal swab is the reference method for detection of SARS-CoV-2 [23, 27], and it is likely that nasal specimens have lower sensitivity than nasopharyngeal specimens [28, 29]. However, we postulate that because of difficulties in obtaining nasopharyngeal samples from children, nasopharyngeal and nasal samples could have similar diagnostic value in children. There is also evidence that nasal samples could be an alternative to nasopharyngeal samples for RT-PCR testing [30] for SARS-CoV-2. Thus, when children show symptoms compatible with COVID-19, one possible option is to use the Panbio™ Test with a nasal sample, because it is non-invasive and better tolerated. However, but a negative Ag-RDT result in a patient who has a high clinical suspicion of COVID-19 should be tested using RT-PCR [9, 31–33].

It is important to highlight that this study mainly examined children in a PHC, and that most of the children who tested positive were asymptomatic or had mild disease. This is contrary to the situation at hospitals, in which most COVID-19 patients have high infectivity and the test sensitivity is therefore higher. For example, Eleftheriou et al*.* (2021) [34] examined 744 children in a tertiary children’s hospital and reported the Panbio™ Test had a sensitivity of 82.35%, and the sensitivity was more that 95% in symptomatic children, greater than in our study or any other study conducted in a PHC [5].

### Strengths and limitations

The main strength of this study was the availability of detailed characteristics of all paediatric participants in a PHC. Secondly, it is important to highlight the high specificity of the Panbio™ test in our population, which greater than 99.6% overall and in the different subgroups. Thirdly, this study has one of the largest samples (*n* = 1142) evaluating Ag-RDTs conducted in paediatric population in PHC. However, there were also some limitations. Firstly, the sensitivity in all 1142 patients was 59.5%, below the WHO recommendation of 80%. Secondly, the study was conducted in a non-vaccinated paediatric population. Thus, even though the vaccination rate is lower in children than adults, our results cannot be extrapolated to vaccinated children. Thirdly, this study was conducted using the Panbio™ Test (Abbott Diagnostics, GmbH, Germany), so caution should be used when applying our results to other Ag-RDTs.

## Conclusion

In our overall paediatric population, the sensitivity of the Panbio™ Test was below the minimum recommended by the WHO, although our specificity met the WHO requirement. Our test sensitivity was greater in children who had symptoms compatible with COVID-19 and were referred by a paediatrician or GP. Although testing a nasopharyngeal swab using an RT-PCR assay is the reference method for detection of SARS-CoV-2, collecting these samples can be difficult in paediatric populations. Our results suggest the accuracy of the Panbio™ test may only be satisfactory in certain subgroups of children or if repeated testing is performed.

## Data Availability

The datasets generated and/or analysed during the current study are available in the Zenodo repository, with the https://doi.org/10.5281/zenodo.7313619 (https://doi.org/10.5281/zenodo.7313619).

## Abbreviations

Ag-RDTs: Rapid antigen-detection tests
CI: Confidence interval
Ct: Cycle threshold
GP: General practitioner
N: Nucleocapsid gene
NPV: Negative predictive value
ORF: Open reading frame gene
PHC: Primary health care centre
PPV: Positive predictive value
RT-PCR: Reverse Transcription-Polymerase Chain Reaction
S: Spike gene
WHO: World Health Organization
